# Supplementary material for: Effects of a joint outdoor exercise program for dog owners and dogs on physical activity, sedentary time and sleep-related behaviors
Source: PLoS One. 2026 Apr 22;21(4):e0346895. doi: 10.1371/journal.pone.0346895 (PMC13102230; doi:10.1371/journal.pone.0346895)
Supplement: S1 Table — Results from Wilcoxon matched-pairs signed rank test comparing baseline and intervention data. All data were not normally distributed, and results are presented as median and min–max values. Significant results are noted in italics. (DOCX) [file pone.0346895.s002.docx]

**S1 Table. Accelerometer-derived sleep-related behaviors in dog owners (n = 15).**

| Sleep parameter | Baseline | Intervention | P-value |
| --- | --- | --- | --- |
| Efficiency (%) | 90.5 (85.7–94.1) | 92.2 (86.1–98.0) | 0.30 |
| Total minutes in bed | 467.5 (369.8–512.8) | 442.6 (348.4–574.0) | 0.60 |
| Total sleep time (min) | 415.7 (343.2–455.7) | 402.0 (320.0–507.0) | 0.93 |
| Wake after sleep onset (min) | 42.0 (23.2–69.2) | 33.4 (10.0–64.3) | 0.11 |
| Number of awakenings | 14.2 (8.2–24.0) | 13.0 (4.4–28.0) | 0.32 |
| Average awakening length (min) | 2.6 (2.0–3.8) | 2.4 (1.4–3.2) | *0.03* |
| Sleep fragmentation index | 22.2 (14.1–40.3) | 19.9 (13.1–41.4) | 0.23 |
| Movement index | 11.4 (8.4–23.9) | 10.7 (8.6–23.6) | 0.45 |
| Fragmentation index | 10.5 (5.4–16.5) | 8.2 (1.3–17.8) | 0.33 |

Results from Wilcoxon matched-pairs signed rank test comparing baseline and intervention data. All data were not normally distributed, and results are presented as median and min–max values. Significant results are noted in italics.
