# Supplementary material for: Effects of a joint outdoor exercise program for dog owners and dogs on physical activity, sedentary time and sleep-related behaviors
Source: PLoS One. 2026 Apr 22;21(4):e0346895. doi: 10.1371/journal.pone.0346895 (PMC13102230; doi:10.1371/journal.pone.0346895)
Supplement: S2 Table — Results from Wilcoxon matched-pairs signed rank test comparing baseline and intervention data. All data were not normally distributed, and results are presented as median and min–max values. (DOCX) [file pone.0346895.s003.docx]

**S2 Table.** **Questionnaire-derived sleep-related behaviors in dog owners (n = 15).**

| Sleep questionnaire | Baseline | Intervention | P-value |
| --- | --- | --- | --- |
| Insomnia Severity Index (ISI) | 7 (1–22) | 6 (0–21) | 0.23 |
| Epworth Sleepiness Scale (ESS) | 5 (0–14) | 6 (0–14) | 0.96 |
| Pittsburgh Sleep Quality Index (PSQI) | 5 (2–13) | 5 (0–9) | 0.28 |

Results from Wilcoxon matched-pairs signed rank test comparing baseline and intervention data. All data were not normally distributed, and results are presented as median and min–max values.
