# Supplementary figures and images for: Effects of a joint outdoor exercise program for dog owners and dogs on physical activity, sedentary time and sleep-related behaviors
Source: PLoS One. 2026 Apr 22;21(4):e0346895. doi: 10.1371/journal.pone.0346895 (PMC13102230; doi:10.1371/journal.pone.0346895)

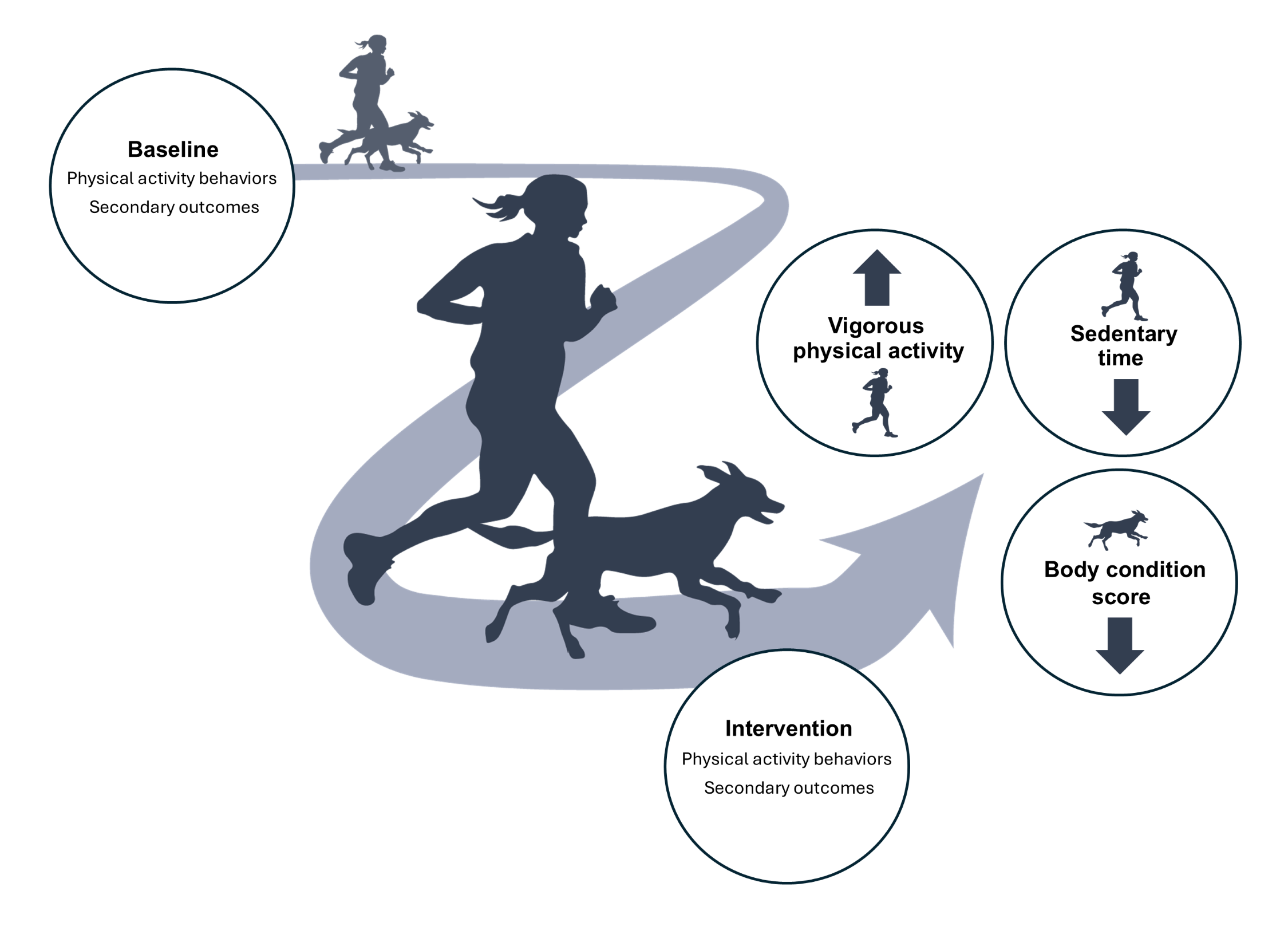

Supplement: S1 Image — (TIF) [file pone.0346895.s004.tif]
