# Supplementary material for: Effects of a joint outdoor exercise program for dog owners and dogs on physical activity, sedentary time and sleep-related behaviors
Source: PLoS One. 2026 Apr 22;21(4):e0346895. doi: 10.1371/journal.pone.0346895 (PMC13102230; doi:10.1371/journal.pone.0346895)
Supplement: Attachment — Submitted filename: Response to Reviewers.docx [file pone.0346895.s006.docx]

**Dear Editor at PLOS One**

Thank you for the possibility to revise and resubmit our manuscript “Effects of a joint outdoor exercise program for dog owners and dogs on physical activity, sedentary time and sleep-related behaviors” (PONE-D-25-61363).

We are most grateful for your constructive criticism and valuable advice, and for giving us the opportunity to revise our manuscript in response. We have carefully tried to meet the concerns and requests from both reviewers, which we believe have resulted in a clear improvement of the manuscript. The point-by-point answers to the Editor and the Reviewers, respectively, are given below.

We hope that these answers along with the revision of the manuscript are satisfactory for publication in PLOS One and look forward to your decision soon.

Sincerely, Klara Smedberg, corresponding author

Swedish University of Agricultural Sciences, Department of Animal Biosciences

Box 7023, 750 07 Uppsala, Sweden

Phone: +46708438377

[klara.smedberg@slu.se](mailto:klara.smedberg@slu.se)

**Point-by-point** **answers to the Editor**

**Journal requirements**

**1. Please ensure that your manuscript meets PLOS ONE's style requirements, including those for file naming.**
*This has been addressed.*

**2. Please state what role the funders took in the study.** 
*The funders had no role in study design, data collection and analysis, decision to publish, or preparation of the manuscript.*

**3. We note that there is identifying data in the Supporting Information file <file name>. Due to the inclusion of these potentially identifying data, we have removed this file from your file inventory. Please remove or anonymize all personal information (<specific identifying information in file to be removed>), ensure that the data shared are in accordance with participant consent, and re-upload a fully anonymized data set.**
*To adhere to the ethical permit and not compromise the privacy of participating dog owners, dog owner raw data is provided in de-identified form. We have uploaded a fully de-identified data set (S1_File). There are no ethical or legal restrictions on sharing this de-identified data set.*

**4. We noted in your submission details that a portion of your manuscript may have been presented or published elsewhere. Please clarify whether this [conference proceeding or publication] was peer-reviewed and formally published. If this work was previously peer-reviewed and published, in the cover letter please provide the reason that this work does not constitute dual publication and should be included in the current manuscript.**
*This manuscript is primarily based on accelerometer-derived data, which is unique and has never been published before. Data on body measurements and some questionnaire-derived data on physical activity/sedentary time included in this manuscript have previously been peer reviewed and formally published elsewhere, but for a larger cohort and with other research questions, aims and results (please see related articles: Smedberg K, Lundbeck E, Roman E, Eriksson JW, Spörndly-Nees S, Kallings LV, et al. A pilot study of a joint outdoor exercise program for dog owners and dogs. Sci Rep. 2024;14(1). doi: 10.1038/s41598-024-65033-0., Söder J, Roman E, Berndtsson J, Lindroth K, Bergh A. Effects of a physical exercise programme on bodyweight, body condition score and chest, abdominal and thigh circumferences in dogs. BMC Vet Res. 2024;20(1). doi: 10.1186/s12917-024-04135-3.). The rationale for including these data in the current manuscript was to fulfill the following aims: 1) to enable comparisons between physical activity/sedentary time as measured by accelerometry, questionnaires and activity diaries, and 2) to assess possible effects of accelerometer-derived physical activity on body measurements. As valid accelerometer and diary data were not provided for all participants in the larger cohort, these aims could only be used in the current study and its smaller cohort of participants. As such, we do not consider the manuscripts to be dual publications.*

**5. We note that you have indicated that there are restrictions to data sharing for this study. PLOS only allows data to be available upon request if there are legal or ethical restrictions on sharing data publicly.**
*The authors confirm that the data supporting the findings of this study are available within the article and its supplementary material. To adhere to the ethical permit and not compromise the privacy of participating dog owners, dog owner raw data is provided in de-identified form. We have uploaded a fully de-identified data set (S1_File). There are no ethical or legal restrictions on sharing this de-identified data set.*

**6. If the reviewer comments include a recommendation to cite specific previously published works, please review and evaluate these publications to determine whether they are relevant and should be cited. There is no requirement to cite these works unless the editor has indicated otherwise.** 
*N/A.*

**7. Please review your reference list to ensure that it is complete and correct. Any changes to the reference list should be mentioned in the rebuttal letter that accompanies your revised manuscript.**
*Please note that two references have been added to the manuscript: Söder J, Roman E, Berndtsson J, Lindroth K, Bergh A. Effects of a physical exercise programme on bodyweight, body condition score and chest, abdominal and thigh circumferences in dogs. BMC Vet Res. 2024;20(1). doi: 10.1186/s12917-024-04135-3 (refence no. 74), Ekelund U, Tarp J, Ding D, Sanchez-Lastra MA, Dalene KE, Anderssen SA, et al. Deaths potentially averted by small changes in physical activity and sedentary time: an individual participant data meta-analysis of prospective cohort studies. Lancet. 2026;407(10526):339–49. doi: 10.1016/s0140-6736(25)02219-6.(reference no. 50).*

**Point-by-point** **answers to the Reviewer comments**

**Response to Reviewer 1**

*We are sincerely grateful for your relevant feedback and constructive suggestions that really have guided us to improve the manuscript.*

**Abstract
- It would be useful to provide a sentence on what kind of exercise regimen you implemented, i.e. describe the methods of the study before listing the results
- Provide data values and p-values for the results you are listing**
*Thank you, these comments have been addressed.*

**Introduction
Line 47: Remove “since decades”
Line 54: You have defined physical activity as PA so use the abbreviation throughout the manuscript
Line 55-57: Replace the instances of “associated to” with “associated with”**
*Thank you, these comments have been addressed throughout the revised manuscript.*

**Methods
Line 76: why were the dogs and humans instructed to wear the accelerometers for different lengths of time (humans 4 days and dog 3 days)?**
*Thank you for pointing out the need for clarification. They were instructed to wear the accelerometer during one week at baseline and one week at the intervention period, but only participants with at least 4 days (humans) or 3 days (dogs) of valid data per period were included in the study. These specific periods of days were based on previous research in each species. This has now been clarified in the revised manuscript.*

**Line 222-225: This sentence is unclear. Why did you multiply by 7 when you had distinct data for 4 separate days? What does 150 respectively 300 min mean? Why did you choose to make each time a criterion when the WHO recommends any time within this range? Why did you choose the minimum and maximum?**
*Since the participants had valid data for less than a week per registration period, we chose to multiply their daily average by seven to enable assessment of fulfilments of WHO recommendations of weekly physical activity time. As the most recent guidelines recommend a weekly range of minutes of PA instead of merely a specified minimum and emphasize the importance of higher levels of MVPA for people with high levels of sedentary time, we chose to assess the proportion of dog owners meeting both the lowest and highest criteria for each recommendation. The sections regarding WHO recommendations have now been revised and clarified in the method section and in the discussion.*

**Line 279: Should be “nearest mm and kg, respectively.”**
*Thank you, this has been addressed.*

**Results
Line 319: So if your total sample size is only 13-15, perhaps a more conservative test of normality should be used like Shapiro-Wilk?**
*Thank you for this valuable suggestion. We have re-analyzed our data with Shapiro-Wilk’s test, which did indeed result in some changes in the distribution of data and therefore also to some changes of method of analysis for detecting changes between registration periods (from t-test to Wilcoxon). Two results that were significant in the first version of the manuscript did not reach statistical significance (P > 0.05), and these were: 1) There was no longer a significant increase in owner-rated perceived physical capacity in dogs (P = 0.06 with Wilcoxon vs 0.03 when t-test was used). 2) When comparing questionnaire-derived PA data with accelerometry there was no longer a significant underestimation of VPA at baseline (P = 0.06 vs 0.03 when t-test was used). We have now revised the relevant sections of the manuscript accordingly.*

**Line 360: It should be 15 and 17%, respectively. I would recheck the entire manuscript for this as I believe you use the term ‘respectively’ quite often.**
*Thank you, this has been addressed.*

**Discussion
Given that the exercise routine that was implemented involved increasing distance and time each week yet the dogs physical exercise did not increase from baseline, do you think this means that the owners did not comply with the exercise regimen? The dogs physical activity should have increased unless they were all already running their end goal distance at baseline right? Especially since this was only measured once at the end where maximal exercise should have been achieved.**
*Thank you for this interesting question, which we also have spent quite some time thinking about when analyzing the data and drafting the manuscript. Based on data from the dog owner questionnaires and activity diaries, it seems reasonable to believe that the vast majority of dog owners did comply with the exercise regimen (although two participants stated that they were not able to reach their selected target distance of 2 km). So, the question is, was there no increase in dogs’ activity levels, or was there an increase that we failed to capture? There may be good arguments for both alternatives. First, it may be reasonable to guess that the dog owners replaced some of their regular dog walks with the jogging sessions, rather than keeping their regular walking habits and adding the exercise sessions on top of that. In Sweden, dogs may be kept off leash under supervision, and most Swedes have access to greenspaces in their local area, which allows time for regular off-leash running for companion dogs. If time in off-leash running was a part of the dogs’ regular walks, exchanging walks for jogging sessions with the owner might not automatically be beneficial for VPA levels in dogs. Furthermore, since most dog owners chose the shortest target distances of 2 or 5 km, it is likely to assume that they were rather unexperienced joggers and that their pace during the jogging sessions was not remarkably high. Thus, the dogs may very well have been able to trot rather than run beside their owners even during the jogging sessions, which then would not have generated any additional time in VPA for dogs. Since the established cut-offs for accelerometry for dogs do not distinguish between walking and trotting, but rather classify them both as LMPA, we do not know if their time in trotting increased on behalf of the time spent walking. However, the fact that their median PA intensity did not change argues against that.*

*On the other hand, it may be possible that PA levels in dogs did in fact increase but were not detected due to methodological constraints, such as insufficient length of the registration periods. The intervention registration period constituted a small sample of the eight-week exercise program and may not have been enough to capture a representative PA pattern for participating dogs during the final week. In addition, it cannot be excluded that the dogs were in fact exercised a bit extra compared to their regular habits when the accelerometers were put on at baseline. In that case, the baseline registrations might have been somewhat exaggerated, and the registrations during the intervention period might in fact represent an increase in PA compared to their true regular habits. Furthermore, the small, but still significant, reduction in BCS (please see further discussion on BCS below) suggests that perhaps something did change in the dogs’ physical activity patterns during the study. To conclude, more studies are needed to evaluate the effect of this exercise program on PA levels in dogs. These should be conducted as randomized controlled trials, using larger cohorts and preferably longer registration periods. We have now tried to elaborate our line of reasoning regarding the lack of changes in PA in dogs in the revised manuscript.*

**Do you think your population of humans was generally more physically active given that a lot of them met the WHO criteria even at baseline. If so, this should be called out as your results may be totally different had you recruited people who were more inactive.**
*Yes, it is indeed plausible that our study group were more physically active than the Swedish dog owner population in general, and that they had a more positive attitude towards exercise as they volunteered to take part in the intervention. This might very well have affected our results, which we now have clarified in the revised manuscript.*

**Similarly, the humans used were generally not overweight so this may explain why you saw no differences in BMI or WHR. Did you look at the data based on baseline BMI? Maybe those who started with a greater BMI did show decreases by the end?**
*On a cohort level, the dog owners had a median BMI of 26.2 at baseline, representing only slight overweight. However, looking at the individual levels, 6/15 dog owners were obese at baseline, and 5/15 at the intervention registration period. Slight decreases in BMI were registered in 10/15 participants, but we did not see greater decreases in more obese subjects.* *We have now specified the information regarding BMI data in the revised manuscript.*

**I think you are missing a big piece of information for the dogs especially but also for the humans and that is diet. I would suspect that even though the dogs were running more as part of the exercise regimen, the human was not adjusting their food intake whereas if the human was doing more exercise, they may be hungrier and eat more for themselves. I think diet needs to be addressed in your discussion relating to the change in BCS but not change in BMI. On the contrary, it could also be explained by the subjectiveness of a BCS in dogs – maybe the change observed was just due to error. Theoretically if the dogs exercise did not change according to your results then their BCS should not change unless the owners were restricting the dogs feed intake which you do not have data on.**
*Thank you, you do absolutely have a point in this. It is quite plausible that the owners might have increased their food intake while keeping their dogs’ usual feeding routines. We have now elaborated the discussion regarding both the food intake and the BCS-reduction in the manuscript. Moreover, the information regarding the BCS-assessments have been clarified in the method sections. All assessments were performed by the same veterinarian, with special expertise in the area. The reduction in BCS is also discussed in relation to results from a previous publication based on a slightly larger cohort from the same intervention, where it was shown that the dogs’ thigh circumference increased significantly while their bodyweight was stable, indicating a redistribution between body fat and muscle mass.*

**Response to Reviewer 2**
*We are deeply grateful for your kind words, and for your valuable feedback that really improved the manuscript.*

**Firstly, I consider the article to be methodologically constrained, primarily due to the small sample size. I acknowledge that conducting a study of this scope, involving multiple assessments that are highly dependent on participant compliance, requires a substantial level of commitment from dog owners throughout the entire project. Nevertheless, the limited sample size may introduce significant bias across the statistical analyses, potentially masking positive findings that may have been observed but did not reach statistical significance (p > 0.05). Although the challenges in maintaining a larger sample are understandable and clearly justified by the author, this limitation reduces the robustness of the results and highlights the need for further studies with larger samples to allow more definitive conclusions.**
*Yes, we completely agree and have tried to stress this more in the discussion and limitations sections.*

**Another point of concern relates to the eight-week evaluation period, which raises questions regarding whether meaningful changes could realistically occur within such a short timeframe and be detected statistically. Given the relevance and alignment of the study objectives, a longer follow-up period (such as six months or even one year) might be more appropriate to capture more definitive outcomes. While extended follow-up presents practical challenges due to participant adherence, this remains an important consideration.**
*Yes, we agree that eight weeks may be too short a timeframe. In this pilot study, we evaluated a pre-existing concept of eight-weeks of joint dog-dog owner outdoor exercise, originally created by the Swedish Working Dog Association. However, with the lessons learned from this intervention, future studies should consider increasing the duration to at least twelve weeks, while also adding follow-ups at six and twelve months.*

**Additionally, the structure of the baseline and intervention periods warrants further clarification. It is unclear why accelerometers were not used throughout the entire eight-week period, as continuous monitoring would have generated a more comprehensive dataset for statistical analysis. Moreover, a single week used as a reference period appears insufficient, as it may represent an atypical week in the routine of the owner and dog. This concern similarly applies to the intervention period, as data appear to have been collected only during the final week of the study. Short assessment windows increase the likelihood of outliers and introduce additional bias. Extending data collection over a longer period would likely provide a more reliable representation of habitual behaviour. Still on the subject of this time period, it was confusing to my understanding: the study lasted eight weeks, but only two weeks of data collection. What were the other six weeks used for?**
*Yes, we agree that continuous monitoring could have captured the participants’ PA behaviors in a more representative way. However, based on previous accelerometer-research in humans we assumed that continuous monitoring might also increase the risk of drop-outs as participants might perceive the accelerometers as somewhat hindering in their everyday life, and would also have to recharge the batteries as needed. Minimum wear time in days for inclusion in the study was based on established recommendations for humans and dogs, respectively. However, with lessons learned from this pilot study, future studies should consider longer periods of data collection. Still, participants were expected to adhere to the exercise program during the entire eight-week period, as we were interested in the effects of the entire period. Dog owners were provided with detailed written instructions for how to increase distance and intensity each week.*

**Regarding the outdoor exercise programme, allowing owners to choose their preferred distance is a positive aspect; however, this approach may also have influenced the statistical outcomes. The absence of significant changes in secondary outcomes, such as BMI and BCS, may be partially attributable to the fact that a substantial proportion of participants selected shorter distances. A discussion or stratified analysis comparing the 2, 5, 7.5 and 10 km groups could have provided valuable insights into the relationship between exercise distance, physical activity levels, sedentary behaviour, sleep patterns, and secondary measures. While subdividing an already small sample presents limitations, the influence of distance travelled warrants consideration.**
*Yes, the small sample size limited the ability to draw conclusions about the effects of the exercise program in relation to workload. To assess the effect of different PA routines/target distances on health outcomes, another study design must be used. The main aim of this specific pilot study was to evaluate the effects of the exercise program on* *PA, sedentary time and sleep-related behaviors, regardless of target distance. The opportunity to individually select a preferred distance may be regarded as a positive aspect that increased compliance, as it gave the dog owners the possibility to participate according to their own and their dogs’ individual capacity.*

**The author notes that exercise distance and intensity were progressively increased throughout the programme; however, it is unclear whether this refers to running sessions, circuit training, or both. Providing further clarification would reduce potential misinterpretation.**
*Thank you for your comment, the exercise program has been described in more detail in the method section in the revised manuscript.*

**Another issue that creates a bias in the assessment: the days of accelerometer use were not standardised. To what extent could this variation (even if only one to two days at most) also influence the final results?**
*The daily mean for each participant was used for calculating cohort-level results, which has been clarified in the method section. While the number of accelerometer wear days was not completely identical across participants, the variation was still limited. At baseline, dog owners wore the accelerometers for a median of 6 valid days (range 5–6 days), and during the intervention period for a median of 5 valid days (range 5–6 days). One weekend day per registration period was included for all participants. Also, mean awake wear time was high and comparable between periods. Dogs wore the accelerometers for a median of 5 valid days at both baseline (range 4–6 days) and during the intervention period (range 4–5 days). Median wear time per 24-hour period was high and comparable between periods, and one weekend day per registration period was included for all dogs, except for one dog that did not provide weekend data at baseline. Given the relatively limited variation in wear days and the consistently high wear time, we consider it less likely to have substantially influenced the final results.*

**With respect to physical activity classification, although WHO guidelines were applied, these classifications rely partly on self-report. As acknowledged by the author, owners tended to underestimate their physical activity levels, introducing subjective bias. Future studies might benefit from prioritising objective measures, such as accelerometer counts per minute, supplemented by owner reports when necessary.**
*Certainly, the need to accelerate the use of device-based approaches to inform a new generation of guidelines has been discussed in previous studies, as it is known that people tend to overestimate their PA levels (even though it turned out that our participant did the opposite!). As such, we chose to assess the degree of fulfilment based on accelerometer-derived PA data. We have clarified this is the method section.*

**The assessment of sedentary behaviour also appears insufficiently described. For owners engaged in predominantly seated occupations, it is unclear how periods of low counts per minute were classified. Similarly, for dogs whose owners work outside the home full-time, opportunities for physical activity may be limited unless deliberately facilitated. Addressing these aspects would strengthen the interpretation of sedentary behaviour.**
*Thank you for pointing this out. We have clarified the definition of sedentary behavior in more detail in the method section (please see Table 1). Hopefully, it is now clearer that most desk-based office work constitute sedentary time. We have also addressed this issue in the discussion. Regarding the dogs; according to Swedish legislation, dogs must be walked at least every sixth hour during the day. As most Swedes work outside the home, many Swedish dogs are either at a daycare center for dogs or are taken out by someone else at least once during the day.*

**Now about the samples, I have some questions about dog owners. First, I think it would be important to highlight and discuss the influence of age on physical activity, because as we age, we humans naturally decrease our exercise and even sleep time. Perhaps the research group could assess whether there were few changes in older humans, if this may have masked the results as well. Finally, regarding the owners, during their mental health assessment, were anxiety or depressive disorders taken into account, for example? It is also known that these are common in the human population and directly affect the ability and amount of exercise.**
*From our understanding, “older humans” in this context is generally defined as people aged 70 years or older. The age of the participants of this study ranged from 23-61. The vast majority (11 participants) were aged 45–64, out of which only two were over 60 years. We saw no tendencies in our data that participants aged 45–64 had fewer changes compared to younger participants. Rather, data showed that the participants overall were physically active and had satisfactory sleep time. We have now clarified the age distribution of the dog owners better in the method section (please see Table 2). Regarding their health status, yes psychiatric disorders such as anxiety or depressive disorders were taken into account but were not reported by the participants. In addition, severe psychiatric disorders were listed as exclusion criteria.*

**Now, regarding the dogs, we must also consider the issue of breed and age. Some breeds have greater ‘energy’ and exercise more easily because they are animals genetically bred for hunting, for example, or with better biomechanics for exercise. In addition, younger animals are also more predisposed to exercise, and this may also influence the results. Again, I understand that the sample was small, preventing the authors from making such assessments, but it is my job to point out this bias and suggest different assessments to be made in a future study (or even discussions to be held in this article).**
*Yes, we agree and regret that it was not possible in this pilot study. We have added a discussion on this to the manuscript.*

**Now, regarding the assessment of dogs' sleep, using whether or not the owner was awakened by their pet during the night as a basis for assessing the dogs' sleep is of little use, as this does not mean that the dog was having a proper, uninterrupted period of sleep, only that it did not go to its owner or that the owner did not wake up with their pet.**
*Yes we agree, the result that most dog owners stated that they never or rarely woke up during the night because of the dog should not be used to assess sleep in dogs, but rather as in indication that co-sleeping with the dog may not have negative effects on the owner’s sleep. It is however consistent with the low nighttime activity levels measured by accelerometry in participating dogs, which indicated no or only very subtle movement. We have added a brief discussion regarding this.*

**Regarding the BCS, how was it performed? Did a single assessor perform the entire BCS for each patient during the reference period and after the intervention period? Was there more than one assessor? Was the assessor experienced? I believe this should be reported because, as we know, although the BCS is an excellent indicator of a dog's body condition, it has a significant flaw in terms of subjectivity, as its qualitative nature can lead to assessor-dependent variations. Therefore, if different assessors were involved during the study or if they were inexperienced, this could explain some changes or even the absence of changes. In addition, the author could discuss precisely this point: that despite the minimal variations in BCS in the study, it is a subjective parameter and that in a short period of time it may not show visible and significant variations to the extent that the assessor can identify a change in score.**
*Thank you for addressing this need for clarification. All assessments were performed by the same veterinarian, with special expertise in the area. The information regarding the BCS-assessments has been clarified in the method sections and is also discussed in relation to our findings. In addition, the reduction in BCS is discussed in relation to results from a previous publication based on a slightly larger cohort from the same intervention, where it was shown that the dogs’ thigh circumference increased significantly while their bodyweight was stable, indicating a redistribution between body fat and muscle mass.*
